# Development and deployment of a field-portable soil O_2_ and CO_2_ gas analyzer and sampler

**DOI:** 10.1371/journal.pone.0220176

**Published:** 2019-08-28

**Authors:** Zachary S. Brecheisen, Charles W. Cook, Paul R. Heine, Junmo Ryang, Daniel deB. Richter

**Affiliations:** Soils Laboratory, Nicholas School of the Environment, Duke University, Durham, NC, United States of America; Universidade Federal de Santa Maria, BRAZIL

## Abstract

Here we present novel method development and instruction in the construction and use of Field Portable Gas Analyzers study of belowground aerobic respiration dynamics of deep soil systems. Our Field-Portable Gas Analysis (FPGA) platform has been developed at the Calhoun Critical Zone Observatory (CCZO) for the measurement and monitoring of soil O_2_ and CO_2_ in a variety of ecosystems around the world. The FPGA platform presented here is cost-effective, lightweight, compact, and reliable for monitoring dynamic soil gasses *in-situ* in the field. The FPGA platform integrates off-the-shelf components for non-dispersive infrared (NDIR) CO_2_ measurement and electro-chemical O_2_ measurement via flow-through soil gas analyses. More than 2000 soil gas measurements have been made to date using these devices over 4 years of observations. Measurement accuracy of FPGAs is consistently high as validated via conventional bench-top gas chromatography. Further, time series representations of paired CO_2_ and O_2_ measurement under hardwood forests at the CCZO demonstrate the ability to observe and track seasonal and climatic patterns belowground with this FPGA platform. Lastly, the ability to analyze the apparent respiratory quotient, the ratio of apparent CO_2_ accumulation divided by apparent O_2_ consumption relative to the aboveground atmosphere, indicates a high degree of nuanced analyses are made possible with tools like FPGAs. In sum, the accuracy and reliability of the FPGA platform for soil gas monitoring allows for low-cost temporally extensive and spatially expansive field studies of deep soil respiration.

## Introduction

Measurement of CO_2_ concentrations in soil profiles is widely utilized to model seasonal fluxes in soil respiration. Although many tools and methods for monitoring CO_2_ are widely used, including field-based manual sampling and real-time data-logging [1–5], there is a deficit in understanding of O_2_-CO_2_ dynamics in *in-situ* soil profile respiration. One important factor to consider in monitoring soil gas dynamics in soil respiration is that CO_2_ is much more soluble in water than O_2_ [6–8]. CO_2_ further undergoes a series of equilibrium reactions that enhances its potential for storage and reactive transport in soil water [3, 7, 9, 10]. Additionally, different SOM (soil organic matter) organic compounds (e.g. organic acids, lipids, carbohydrates) have different metabolic oxygen requirements for respiration [11, 12]. The careful measurement of O_2_ and CO_2_ simultaneously, along with local meteorological data and soil profile chemistry information (e.g. presence or absence of carbonate minerals), can be leveraged to account for these factors via calculation and interpretation of the Apparent Respiratory Quotient (ARQ) [7]. While CO_2_ is frequently measured in soil respiration studies, O_2_ measurements are frequently missing from soil monitoring setups. When used, O_2_ sensors are often buried in the soil and subjected to environmental stresses of diurnal and annual temperature and moisture fluctuations with potential for sensor measurement drift through time [1, 7, 13, 14]. Though there are products on the market targeted for portable field use that measure both CO_2_ and O_2_ via aspirated flow-through analysis, they are may be prohibitively expensive for researchers, often in excess of 20,000 USD. Existing commercial products are also frequently quite heavy, in excess of 15kg without an internal battery supply (which may add an additional 10kg or more), reducing actual field portability. Lastly, existing products often do not offer native capacity for the collection of gas samples in the field for laboratory analyses. In order to address the problems outlined above, we present a field-portable gas sampler and analyzer (FPGA) platform which is less expensive to construct, light weight, and robust in its performance.

The FPGA platform presented herein measures O_2_ and CO_2_ concentrations in percent (%) units and can be constructed from materials totaling approximately 2,000 USD and weighs less than 10kg, including internal battery power. Though absolute changes in gas concentrations of soil O_2_ and CO_2_ compared to above ground ambient concentrations are generally very similar, the relative change in concentration of CO_2_ belowground compared to aboveground spans at least two orders of magnitude. Concentrations of soil pore space CO_2_ are generally 10 to 200 times higher than they are in the above ground atmosphere (~0.04%), reflecting reductions in O_2_ belowground relative to the aboveground ambient concentration of approximately 20.95% [7, 9, 15]. Because soil CO_2_ values are so high, measurement precision at the level of parts-per-million (ppm, 1ppm = 0.0001%), as reported in atmospheric eddy-covariance studies, may not be necessary depending on the research topic and the magnitude of ecological, abiotic, or treatment effects on soil systems [3, 6, 16–18]. The FPGA reliably and robustly allows for the measurement of gaseous O_2_ and CO_2_ with at least 0.1% measurement precision for both gasses.

## Materials & methods

### Field installation of buried soil atmosphere gas reservoirs

**Fig 1:**
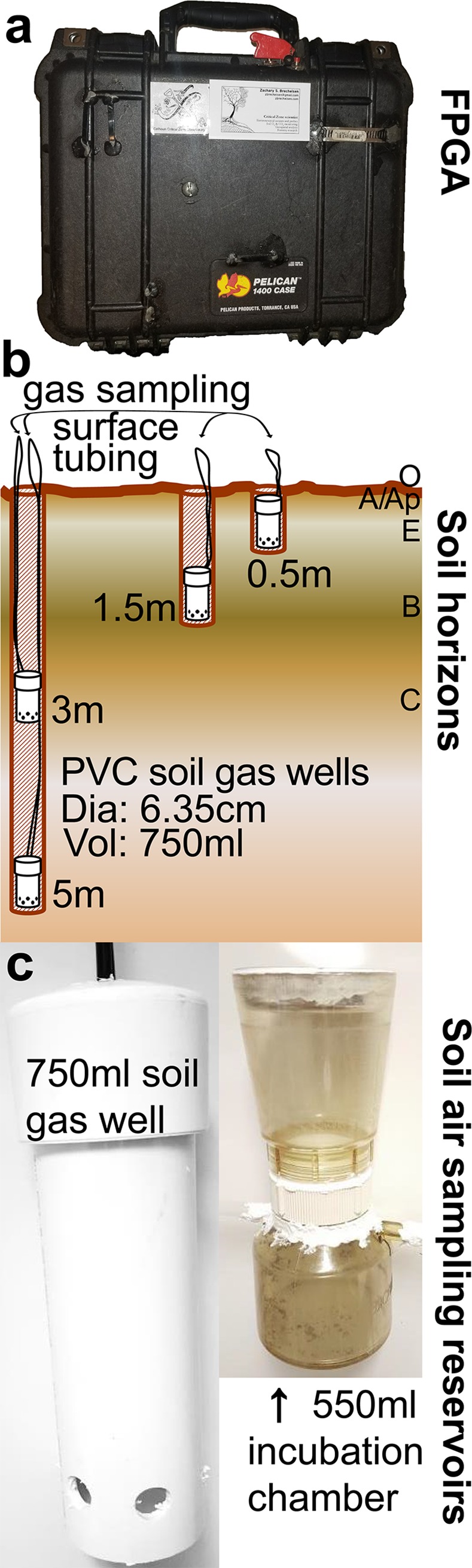
Soil atmosphere monitoring equipment and installation diagram. a) Closed FPGA for transport. b) Field installation diagram of soil atmosphere chambers. c) PVC-constructed gas well (left); and soil incubation chamber (right).

In order to conduct *in situ* measurements of soil gasses using an FPGA (Fig 1A), it is necessary to install soil gas reservoirs (i.e. “gas wells”) into the soil profile (Fig 1B and 1C). This is accomplished by: 1) using a 10cm diameter auger to reach the desired soil depth or until refusal, keeping the excavated soil sorted into known depth increments 2) The careful lowering of a 750mL (smaller volumes are possible depending on soil conditions) gas well constructed from PVC pipe to the bottom of the borehole using the gas sampling Bev-A-Line XX tubing cut to a length equal to the borehole depth plus ~50cm for each of the two sampling tubes inserted into the top of soil gas wells. 3) Joining the two ends of the sampling tubes using a compression fitting, labeled with the soil depth at the bottom of the gas well, pulling it to attain slight tension on the tubing, then securing the joined sampling tubes to the soil surface adjacent to the borehole. The partial tension on secured sample tubes allows for backfilling of depth-appropriate soil into the borehole to be re-compacted without exposing the sample tubing to crushing during compaction. 4) With the gas chamber lowered into the borehole, the careful re-filling a small amount (~2L for a 750mL chamber) of depth-appropriate soil without re-compaction. This is done to prevent crimping or pinching of gas sampling tubes coming out of the top of the gas wells and because it maximizes local gas permeability in the volume around the gas well. If using a different diameter auger or different size or shape chamber, more or less initial soil may be needed to completely surround and cover the gas well at the bottom of the borehole, so researchers should adjust the soil volume accordingly. Initial soil filling volume can be determined or verified via shallow gas well installations as depths where chambers and tubing are easily visible inside the borehole when looking down from the surface. Add known volumes of soil until the entire chamber is covered plus ~10cm above it keeping track of the volume sum. 5) Slowly continue to pour depth-appropriate soil back into the borehole but now compressing the soil as it is filled in. This can be efficiently accomplished using a small flat disk on the end of a long pole to compress and tamp back-filled soil as it is poured into the borehole. We have used bulk density sampler caps attached to the auger extension poles successfully for this purpose. 6) In particularly deep or difficult to work soils (e.g. very stony or clay-rich) or if time in the field is limited, it can be advantageous to stack gas well installations as in Fig 1B. Follow the same procedures as in steps 2–5, but be especially careful to make sure the bottom of the partially re-filled bore hole is at the desired depth from the soil surface and that the back-filled soil has been very well compacted to minimize gas diffusion, and advection during pump sampling, from other depths in the back-filled borehole. For shallower gas well installations, we recommend separate and individual gas well installations to minimize the local soil disturbance below each gas well.

Gas wells are installed in order to draw soil profile air to the soil surface for measurement and sampling. Buried reservoirs we have used in the field have ranged from 250ml to 750mL in volume. High volume gas wells may be preferable to increase the volume of soil profile air analyzed and sampled, though some circumstances necessitate smaller gas wells. The reservoirs are open on the bottom and capped at the top (Fig 1C) and are installed vertically in the soil. Horizontal gas chambers have been used at shallow depths in agricultural settings where plowing was a concern, though horizontal installation is more laborious has not been used outside of agricultural sites. A single length of Bev-A-Line XX tubing is inserted through a pair of drilled openings in the PVC cap to form a loop. With proper fit and using care not to pull on the tubing as the augered hole is backfilled, no adhesive or adapter is required to secure the tubing around the openings. The tubing is then used to lower the reservoir to its designated depth. After the auger hole is completely backfilled, the closed end of the loop will remain above the soil surface. A spare PVC reservoir placed on the soil surface can offer some protection for emergent tubing from rodents. A JACO nylon or Kynar compression fitting union (6.35mm ID) creates an airtight junction in the loop where the FPGA can be connected for sampling and measurement purposes. The design and installation of gas well reservoirs do not directly influence the operation of the FPGA. Newly installed reservoirs may require several days to equilibrate with the surrounding soil air, depending on depth and soil texture. Equilibration can be accelerated by pumping many liters of air out of the gas well (not recirculating it) for several minutes after backfilling and compaction is complete.

### Design and construction of the FPGA

**Fig 2:**
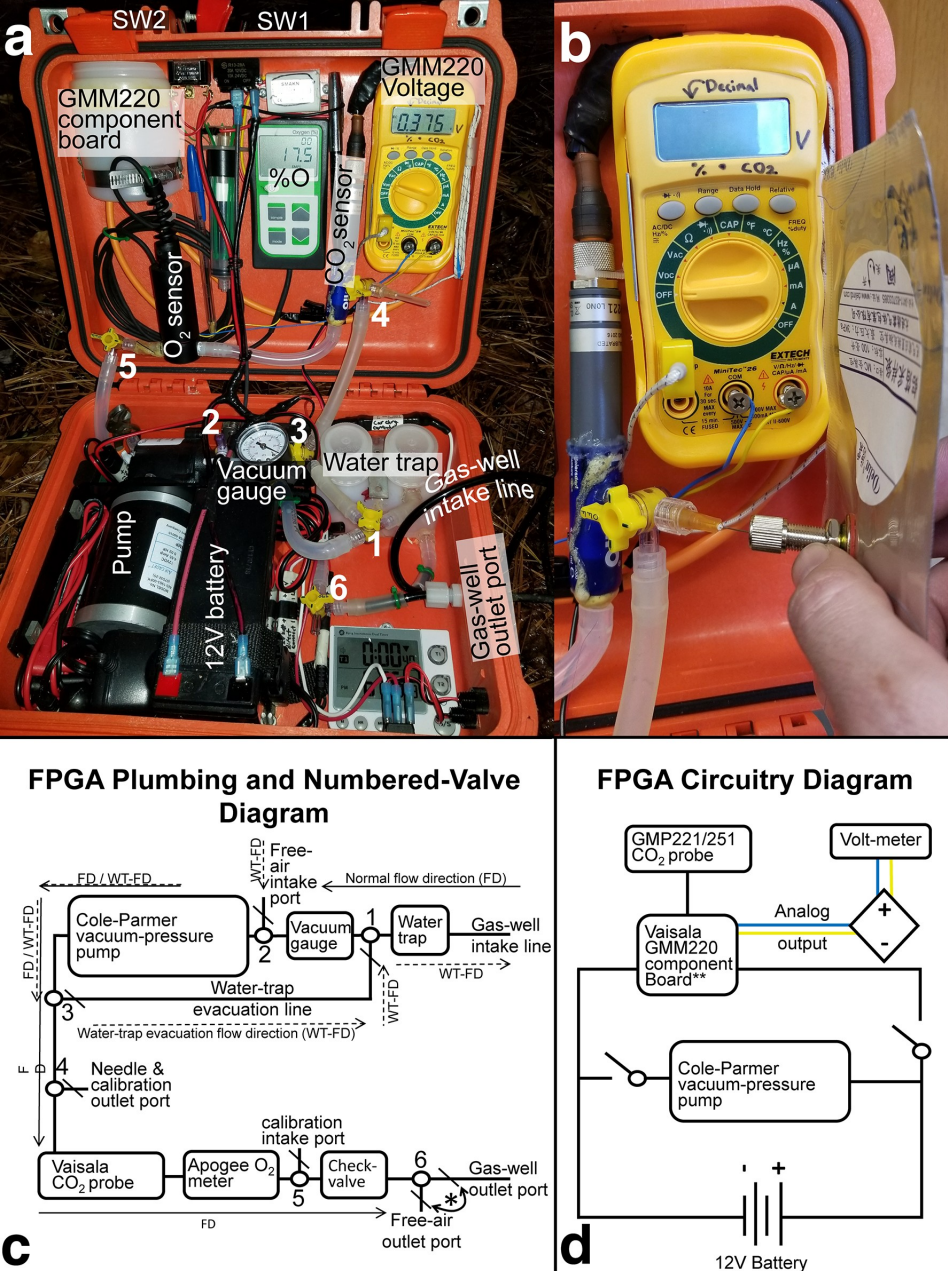
Annotated FPGA components, plumbing, and wiring. a) annotated FPGA components as seen during field deployment, b) closeup view of hypodermic needle filling a gas sample collection bag, c) plumbing diagram of the FPGA indicating flow directions through different components of the FPGA for regular use as well as water-trap evacuation. Numbered 3-way valves with “\” indicating the line is closed during normal use. d) Circuitry wiring diagram for the FPGA.

The FPGA platform integrates several off-the-shelf components to form a robust and reliable system for field O_2_-CO_2_ measurements, see Table 1 for complete parts list. Housed within a Pelican model 1400 case (Figs 1A and 2A), the platform is comprised of three main components: Cole Parmer Air-Cadet vacuum-pressure pump (model EW-07532-25); Vaisala GMP 221 CO_2_ probe with GMM220 transmitter module (the GMM220 series has recently been replaced by the GMP251 model, which still offers analog voltage data output), and an Apogee MO220 O_2_ meter (Fig 2A). The Apogee O_2_ meter can be ordered with a flow-through head installed, but a flow-through adapter for the CO_2_ probe must be ordered from Vaisala or manufactured by the user. A 7Ah 12V battery (Fig 2A and 2D) is used to power the Cole-Parmer pump and Vaisala probe. The pump and probe have dedicated power switches. Under normal operating conditions, a fully charged battery can power both devices for a full day of sampling. The Apogee oxygen meter and digital voltmeter have internal battery power.

**Table 1:** Itemized component list for the construction of a Field Portable Gas Analyzer.

*Supplier*	*Part number*	*Description*	*Quantity*	*Unit price (USD)*	*Website URL*
**FPGA materials**					
Amazon	1270	12V 7ah battery	1	22.35	http://www.amazon.com/gp/product/B002BJU8YQ
Apogee	SO-220	Handheld Apogee oxygen meter	1	426	https://www.apogeeinstruments.com/mo-200-oxygen-sensor-with-handheld-meter/
Apogee	AO-002	Flow-through adapter	1	36	https://www.apogeeinstruments.com/oxygen-meter-sensor-flow-through-head-ao-002/
Bjerg Inst.	NA	Timer	1	19.99	https://www.amazon.com/gp/product/B01BUCOG60
Cole-Parmer	EW-07532-25	Air cadet pump	1	350	https://www.coleparmer.com/i/mn/0753225
Cole-Parmer	EW-30600-23	Large-bore 3-way, male-lock, stopcocks	1	35.05	https://www.coleparmer.com/i/cole-parmer-large-bore-3-way-male-lock-stopcocks-10-pack-non-sterile/3060023?searchterm=EW-30600-23
Cole-Parmer	EW-30800-06	Female Luer to 1/4" J Barb Adapter, 25pk	1	11.8	https://www.coleparmer.com/i/cole-parmer-adcf-female-luer-to-1-4-j-barb-adapter-pp-25-pk/3080006?searchterm=EW-30800-06
Cole-Parmer	EW-30800-22	Male Luer to 1/4" J Barb Adapter, 25pk	1	17.9	https://www.coleparmer.com/i/cole-parmer-adcf-male-luer-to-1-4-j-barb-adapter-pp-25-pk/3080022?searchterm=EW-30800-22
Extech	MN26T	Extech MN26T Multimeter	1	44.99	https://www.amazon.com/gp/product/B0000WU1AM
Grainger	2GUL4	female branch tee	1	8.17	https://www.grainger.com/product/PARKER-Barbed-Female-Branch-Tee-2GUL4?searchBar=true&searchQuery=2GUL4
Grainger	4FLZ2	Vacuum gauge	1	13.46	https://www.grainger.com/product/GRAINGER-APPROVED-1-1-2-Test-Vacuum-Gauge-4FLZ2
Pelican	1400	Pelican 1400 Case	1	77.95	https://www.amazon.com/Pelican-1400-Case-Camera-Black/dp/B00009XVKY/
Pilot Auto.	PL-SW26	Toggle switch	1	6.37	https://www.amazon.com/gp/product/B000GTMUUI
US Plastic Corp.	61057	Jaco 1/4" nylon bulk-head union	1	1.37	https://www.usplastic.com/catalog/item.aspx?itemid=29652
Vaisala	GMP251A5A0A0N1	GMP251—CO2 probe	1	702	https://store.vaisala.com/us/gmp251-co2-probe-for-level-measurements-voltage-output-current-loop-modbus/GMP251A0A0A0N1/dp
Vaisala	ASM211697SP	Flow-through adapter with gas ports for GMP251	1	67	https://store.vaisala.com/us/flow-through-adapter-with-gas-ports-for-gmp251/ASM211697SP/dp?refSrc=GMP251A0A0A0N1&nosto=right-block
Vaisala	223263SP	Probe cable (1.5m) with open wires for Indigo probes	1	41	https://store.vaisala.com/us/probe-cable-with-open-wires-1.5-m-for-indigo-compatible-probes/223263SP/dp?refSrc=GMP251A0A0A0N1&nosto=right-block
Amazon	NA	One-way check valve	1	8.8	https://www.amazon.com/gp/product/B06XK2C13R/ref=oh_aui_search_detailpage?ie=UTF8&psc=1
Amazon	Morris 70270	Power switch for probes	1	8.39	https://www.amazon.com/gp/product/B005GDG2M6/ref=oh_aui_search_detailpage?ie=UTF8&psc=1
Masterflex	L/S 24, 25 ft	Platinum-cured silicone tubing	1	138	https://www.masterflex.com/i/masterflex-platinum-cured-silicone-tubing-l-s-24-25-ft/9641024?pubid=SI
**Total (USD): 2036.59**					
**Soil gas well connections and tubing**					
US Plastic Corp.	56285	Bev-a-line XX Tubing .170" ID x 1/4" OD	TBD	0.6/ft	https://www.usplastic.com/catalog/item.aspx?itemid=35641
US Plastic Corp.	61005	Jaco 1/4" nylon union	TBD	0.92	https://www.usplastic.com/catalog/item.aspx?itemid=25575

Inside the FPGA, Masterflex Platinum Tubing (L/S 24) is used for all air-flow connections because it tolerates repeated bending and flexing without forming kinks. To prevent soil water from entering the system, in-line water traps are installed. Water traps can be fashioned from a variety of vessels. They are designed such that inflow enters the bottom of the trap and outflow occurs towards the top. Water traps with a volume of 250ml or greater should give users enough time to turn off the pump if saturated soil conditions are encountered. To provide warning of flooded wells and avoid stress or damage to the system, a vacuum gauge with a range of -100:0 kPa is used inline before the pump. If the input line is clogged, kinked, or flooded, the vacuum pressure will drop below -20kPa and the user can shut off power to the pump.

With the pump on, soil pore space air flows through the Vaisala and Apogee meters, providing nearly instantaneous readings of CO_2_ and O_2_. A 1-way check-valve is installed before 3-way valve number 6 (Fig 2A and 2C) to prevent backward flow or diffusion of aboveground air into the system during measurement. Numbered 3-way valves are used to direct and control air flow pathways. This gives the FPGA capability for flow-through gas analysis, circulating gas analysis, or sample collection via hypodermic needle (e.g. 26ga). If water is drawn into the water trap, the flow direction of pumped air can be reversed (after turning the pump off) in order to empty the water trap (Fig 2B). After valve 6 (Fig 2C) a short segment of Bev-A-Line tubing (6.35mm OD) is inserted into the end of the Masterflex tubing and connected to a JACO nylon bulk-head junction (6.35mm ID) for connection to the soil gas well outlet port. Bev-A-Line XX tubing was specifically chosen for its low gas permeability.

### FPGA field deployment

Six FPGAs have been constructed over the last 6 years. They have been used to monitor soil O_2_ and CO_2_ dynamics in the Southern Piedmont of the US, hyper-arid Peruvian deserts, and the eastern foothills of the Pfälzerwald mountains in southwestern Germany. Most measurements were confined to the upper 5m of soil (Fig 1B). In two field locations in the United States, measurements have been extended to 8.5m belowground and sampled successfully many times when the groundwater table was sufficiently low. In Germany, a gas well reservoir was installed and sampled at 10m depth. Sampling has taken place in a variety of remote field settings including agricultural fields and orchards, managed timber pine forests, natural hardwood forests, and vineyards. Field data presented herein were collected in the Enoree district of the Sumter National forest with permission from the US Forest Service, the South Carolina Department of Natural Resources, and private landowners. The locations of research plots are presented in Table 2.

**Table 2:** Soil atmosphere sampling plots at the CCZO.

Plot_name	latitude	longitude
R1_C1_0.5	34.61014	-81.727
R1_C1_1.5	34.61014	-81.727
R1_C1_3_5m	34.61017	-81.727
R1_C2_3_5m	34.61147	-81.7279
R1_C3_0.5_3_5m	34.60924	-81.728
R1_C3_1.5	34.60925	-81.728
R1_H1_0.5	34.60642	-81.7233
R1_H1_1.5	34.60642	-81.7234
R1_H1_3_5	34.60642	-81.7233
R1_P1_0.5	34.60741	-81.7228
R1_P1_1.5	34.60742	-81.7228
R1_P1_3_5	34.60739	-81.7228
R1_P2_0.5_1.5_3_5	34.60811	-81.7225
R1_T1_0.5	34.61041	-81.7187
R1_T1_1.5	34.61045	-81.7186
R1_T2_3_5_8.5	34.61053	-81.7177
R1_T2_3_5	34.61059	-81.7176
R1_T_0.5	34.60979	-81.7181
R1_T3_1.5	34.60979	-81.718
R1_T3_3_5	34.60982	-81.718
R1_T4_3_5	34.61054	-81.7197
R1_T5_0.5	34.60912	-81.7181
R1_T5_1.5	34.60914	-81.7181
R1_T5_3_5	34.60906	-81.7181
R1_T6_1.5	34.61004	-81.7188
R1_T6_3_5	34.61008	-81.7188
R1_T7_3_5_8.5	34.6097	-81.7195
R4_H1_0.5	34.59827	-81.6758
R4_H1_1.5	34.5982	-81.6759
R4_H1_3_5	34.59824	-81.6758
R4_P1_0.5	34.5988	-81.6838
R4_P1_1.5	34.59882	-81.6838
R4_P1_3_5	34.59877	-81.6838
R7_H1_0.5	34.54218	-81.7549
R7_H1_1.5	34.54212	-81.7549
R7_H1_3_5	34.54215	-81.7548
R7_P1_0.5	34.54145	-81.7555
R7_P1_1.5	34.54147	-81.7555
R7_P1_3_5	34.54152	-81.7555

Measurement of O_2_ and CO_2,_ concentrations is accomplished in the following steps:

1) Powering on the CO_2_ and O_2_ meters (switch 2) and running the pump (switch 1) for 30–45 seconds with nothing attached to the FPGA intake and outflow ports (Fig 2A, 2C and 2D). This is done in order to calibrate the Apogee oxygen meter to ambient atmospheric conditions, assumed to be 20.95% O_2_. The user should also check for proper functioning of Vaisala CO_2_ probe and allow it to warm up and stabilize for approximately 1 minute before soil gas measurement, as recommended by Vaisala. The CO_2_ probe should return an analog voltage signal read via a voltmeter (Fig 2A, 2B and 2D) equivalent to approximately 0.04% CO_2_. Actual analog voltage reported by the GMM220 series component board is dependent on the configuration ordered from Vaisala. Specific voltage multipliers (e.g. if 1V = 2% CO_2_, %CO_2_ = 2*V_analog_) may be required depending on the requested specifications. A direct 1V = 1% CO_2_ or 0.1V = 1%CO_2_ are the most convenient configurations for data collection in the field.
2) Connect the gas reservoir lines to the intake and outflow ports (Fig 2A and 2C).
3) Flush the FPGA system with 1L or more of soil gas (30–45 seconds) to saturate the entire system volume with soil atmosphere, having valve 6 closed towards the gas-well outlet port and open to the free-air outlet port (Fig 2A and 2C).
4) After flushing a sample may be collected for laboratory analysis by turning valve 4 to needle outflow (valve 4 “off” should be pointing towards the CO_2_ probe line) (Fig 2A, 2B and 2C).
5_1_*) for non-circulating gas measurement, turn off power to the pump using switch 1 (Fig 2A and 2D) and record the maximum concentration value observed for both CO_2_ and O_2_ within 1 minute. There is a protective PTFE sleeve inside the outer shell of the GMP221 CO_2_ probes that protects and shields the NDIR sensor, but it also slightly delays equilibration of pumped soil gas. Because of this, it is normal to observe a gradual increase in reported CO_2_ concentration to a peak, typically followed by a gradual decline. This pattern assumes gas well sampling progresses from shallower to deeper depths, as soil CO_2_ concentrations tend to increase with depth up to 3–5 meters.
5_2_*) If it is necessary to evacuate a smaller volume of gas from the soil gas reservoir and surrounding soil volume, the user may limit step 2 to flushing the system for a shorter period of time (at least 10 seconds is necessary to flush the water trap, pump, tubing, and probe volumes) and then close valve 6 towards the free-air outlet port and opening it towards the gas-well outlet port in order to conduct circulatory gas analyses. We have observed an apparent dilution effect of up to a 0.1–0.2% CO_2_ decrease using this gas circulating methodology and so prefer the 5_1_* procedure. Procedure 5_2_* may be necessary, however, when sampling very low diffusivity saprolite or soils with very high clay content when partially saturated with water. 6) After sampling is complete at a site, and if the time until the next measurement will be taken is more than 10 minutes, it is recommended to turn off power to the Vaisala GMM220 (switch 2) until the next sampling, as it will draw down battery power considerably if left turned on consistently.

In addition to in-situ gas analysis, the FPGA can be used to collect samples for laboratory analysis. This is accomplished with inflatable sample bags made of metalized plastic film or Tedlar with 100ml or greater capacity. Sample is injected through a septum using a 26-gauge hypodermic needle mounted on valve 4 (Fig 2B). Tests have shown these bags to keep samples stable for up to 3 days. For longer storage, samples should be transferred to evacuated glass vials using a closeable syringe. FPGAs are also suitable for bench-top applications including soil incubation experiments (Fig 1C) using small circulating hand pumps widely available like those offered by Mityvac.

### Measurement validation, data plotting, and calculation of Apparent Respiratory Quotient (ARQ)

Measurement validation was accomplished by measuring *in situ* CO_2_ and collecting field samples every three weeks from 31 September 2015 to 16 August 2017 for laboratory analysis. Soil atmosphere gas samples were analyzed for CO_2_ concentrations on a Varian 450-GC gas chromatograph. Data analysis and plotting was conducted using R statistical software [19] with the fields package [20]. In addition, the FPGA was tested periodically by measuring O_2_ and CO_2_ in certified reference gases. Testing revealed a faulty Apogee MO120 meter that was replaced in early 2016. For O_2_ concentrations below 15%, the meter was reporting values consistently lower than the documented value. Because O_2_ concentrations below 15% have been rare in the upland terrestrial systems studied thus far, equipment error is not believed to have significantly biased data from this period.

Timeseries datasets of gas concentrations measured across multiple depths can be powerfully plotted and interpreted using heatmap plots [4, 6]. Plots presented here were generated using the “fields” package in R [19, 20] using the Krig function (covariance = “Matern”, theta = 10, smoothness = 1) for kriging interpolation of the Z-axis gas concentration values which are colored in the heatmaps. The Krig object can be queried and plotted for quality assurance that the interpolated values closely match the original data. Soil sampling depth is plotted on the y-axis and date is plotted along the x-axis. Date was converted to a consecutive integer format and scaled to be of similar range to values in the y and z axes (~0–10) prior to kriging. After kriging, the surface plotting function was used with type =“C” in order to plot contour lines in addition to colored heatmap plotting.

While both O_2_ and CO_2_ timeseries concentration data are very valuable individually, they can be integrated through the calculation of the Apparent Respiratory Quotient (ARQ) which is calculated as the increase in soil CO_2_ above ambient atmospheric conditions (0.04%) divided by the reduction in soil O_2_ below ambient atmospheric conditions (20.95%) and then multiplied by a diffusivity coefficient (0.76) [7]. The equation is thus:

$$Soil\;ARQ=0.76*(\frac{\Delta CO2}{\Delta O2})$$

The soil ARQ will tend to equal approximately 1 assuming a carbohydrate substrate for autotrophic and heterotrophic respiration [11] and also assuming no other biotic or abiotic sources or sinks for either O_2_ or CO_2_ in the ecosystem. Both of these assumptions are frequently violated [7] which makes the ARQ a valuable tool for linking soil gas dynamics to soil organic matter quality, soil moisture and infiltrating precipitation, and the nature of redox-active soil minerals.

## Results

### FPGA platform validation

**Fig 3:**
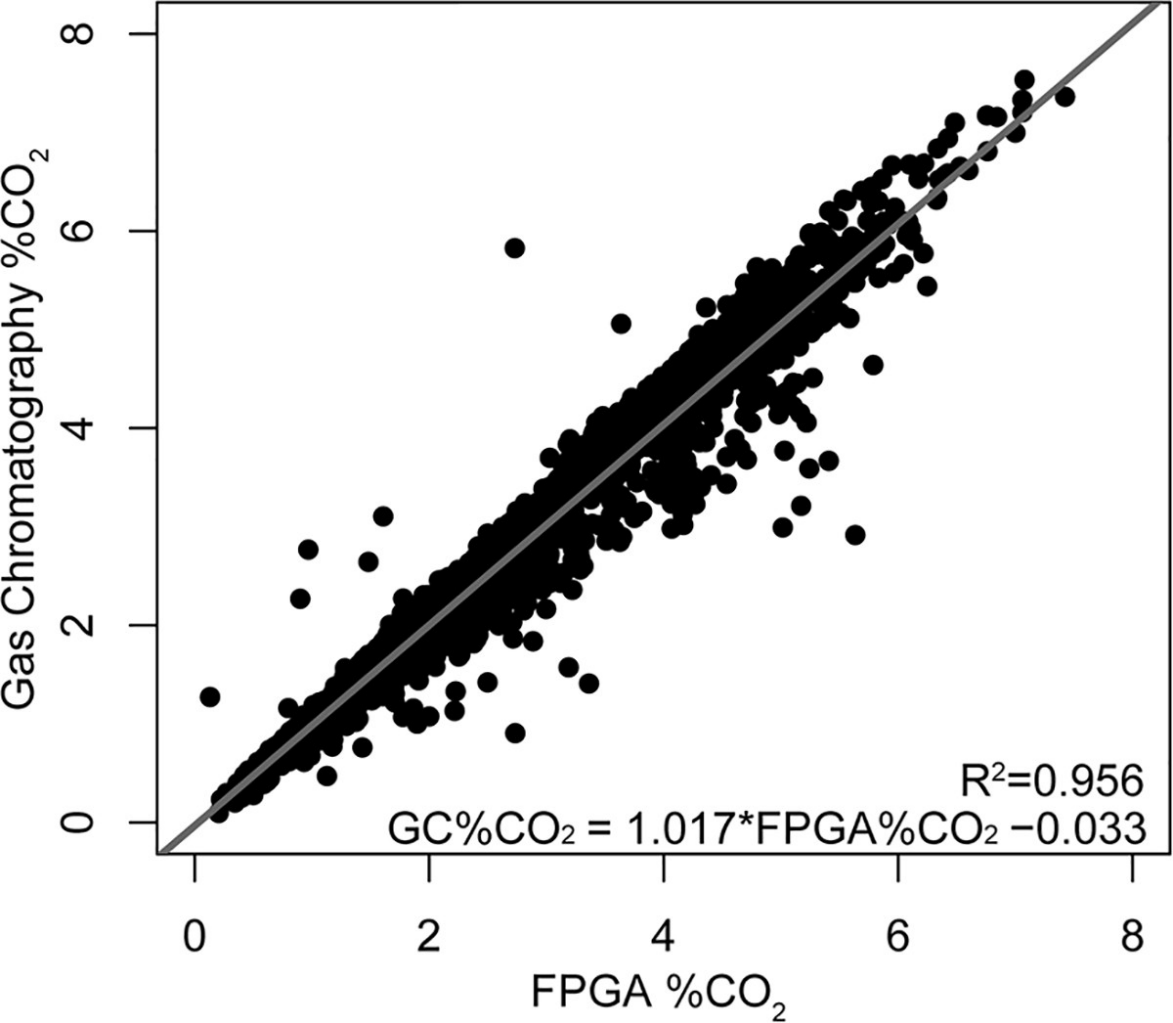
FPGA CO_2_ measurement validation. Carbon dioxide measurement comparison of 1639 observations between FPGA and laboratory gas chromatography. Linear regression results are in the bottom right. Observations are pooled among soil depths and landcover types including hardwood forests, agricultural fields, and mixed pine forests. The grey 1:1 line represents equivalent CO_2_ concentrations between field (x-axis) and laboratory (y-axis) measurements.

Validation of FPGA CO_2_ measurement accuracy was evaluated via 1639 parallel field and laboratory measurements, which indicate high agreement and reliability (Fig 3). A linear regression of the two datasets results in a slope close to 1 at 1.017 and an intercept close to 0 at -0.033. Further, an R^2^ of 0.956 indicates very high agreement between the field and laboratory measurements of CO_2_ and high reliability of the FPGA for measurements within the observed ranges of ~0.25–7.75% CO_2_ measured in field soils.

### Temporal observations and Apparent Respiratory Quotient

**Fig 4:**
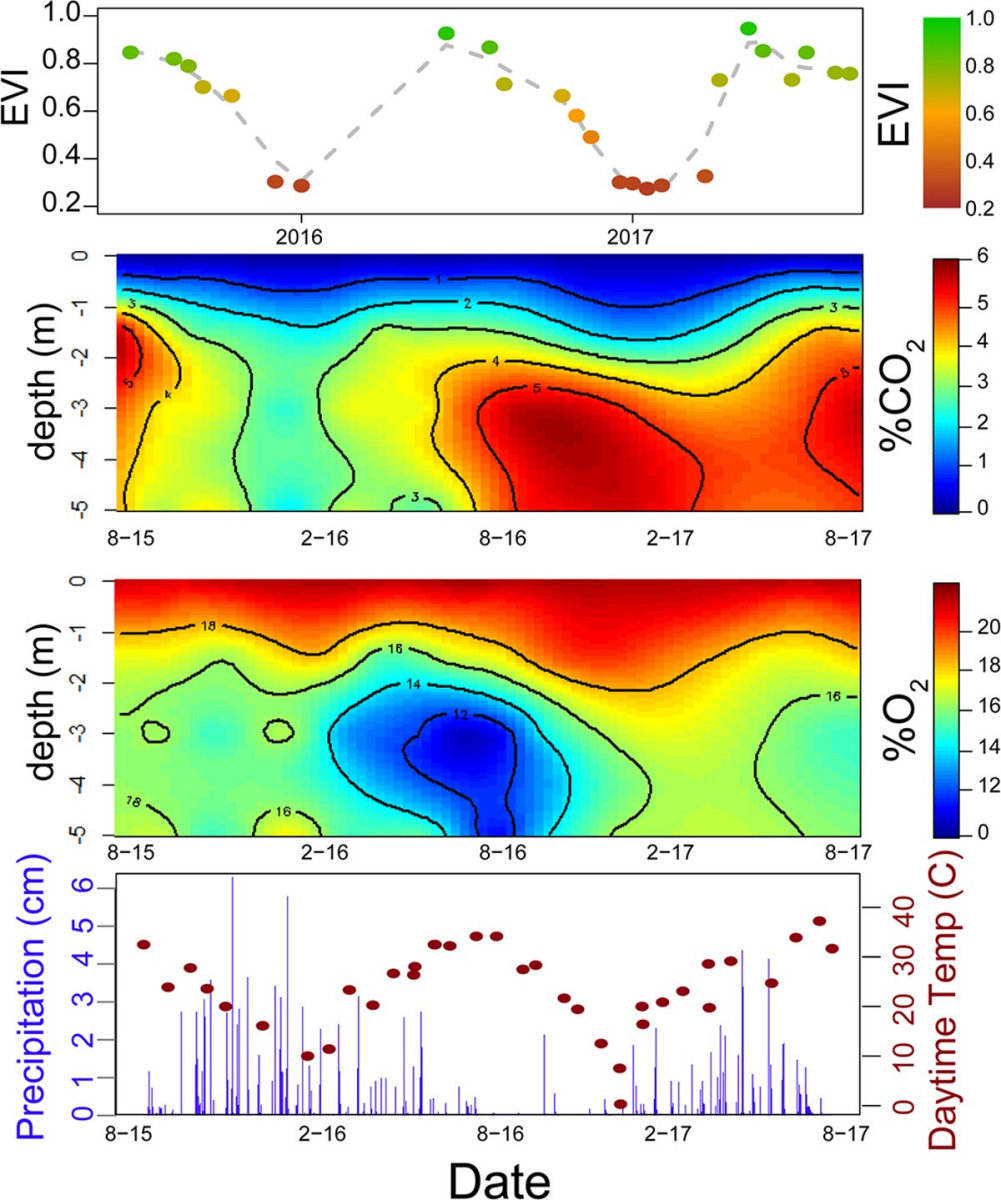
Graphical presentation of soil gas and climate data. Time series heatmap plotting of (top row) plot-averaged (n = 3) hardwood forest [23] Enhanced Vegetation Index (EVI) values, where higher/greener values indicate greater photosynthetic leaf area during spring and summer with declines during fall and winter [21]. EVI raster data derived from Landsat 7 data were downloaded using Google Earth Engine Explorer [22]. FPGA-measured CO_2_ and O_2_ averaged across three replicate hardwood forests plotted via heatmaps (middle two rows) represent high gas concentrations in red and low concentrations in blue. FPGA-measured aboveground temperature and NOAA precipitation are plotted in the bottom row. Black points correspond to mean daytime temperature during field sampling and grey bars indicate total daily precipitation. Precipitation data are from a weather station in nearby Spartanburg, SC. Date is on the x-axis on all plots.

Time series plotted via heatmaps highlight some seasonal dynamics in studies at the Calhoun CZO of soil respiration in hardwood forest soils (Fig 4) in both the accumulation of CO_2_ and the decline of O_2_ during warmer spring and summer months with high Landsat-derived EVI and temperature values. These patterns are reversed in the fall and winter, as EVI and temperature decrease (Fig 4). Temperature values are day-time averages observed over the course of gas-sampling and measured via a thermocouple connected to the multimeter used to read the CO_2_ voltage. Publicly available Landsat-derived Enhanced Vegetation Index (EVI) [21] timeseries data were obtained via Google Earth Engine Explorer [22]. Averages of 30m resolution EVI raster data across three 120m radius hardwood plots [23] were averaged using ArcGIS and R geospatial software [19, 24, 25]. There is an apparent decline in soil CO_2_ concentrations during high precipitation time periods, which is not reflected by an increase in soil O_2_ when plotted alongside publicly available precipitation data from Local Climatological Data (LCD) (WBAN: 93804) via NOAA’s National Climate Data Center [26]. Precipitation data were downloaded at 20 minute-interval resolution, converted to mm, and aggregated into daily totals. This deviation from a 1:1 relationship between the two gasses is likely due the greater solubility of CO_2_ relative to O_2_ and a series of equilibrium reactions generating carbonic acid and bicarbonate [6–8].

**Fig 5:**
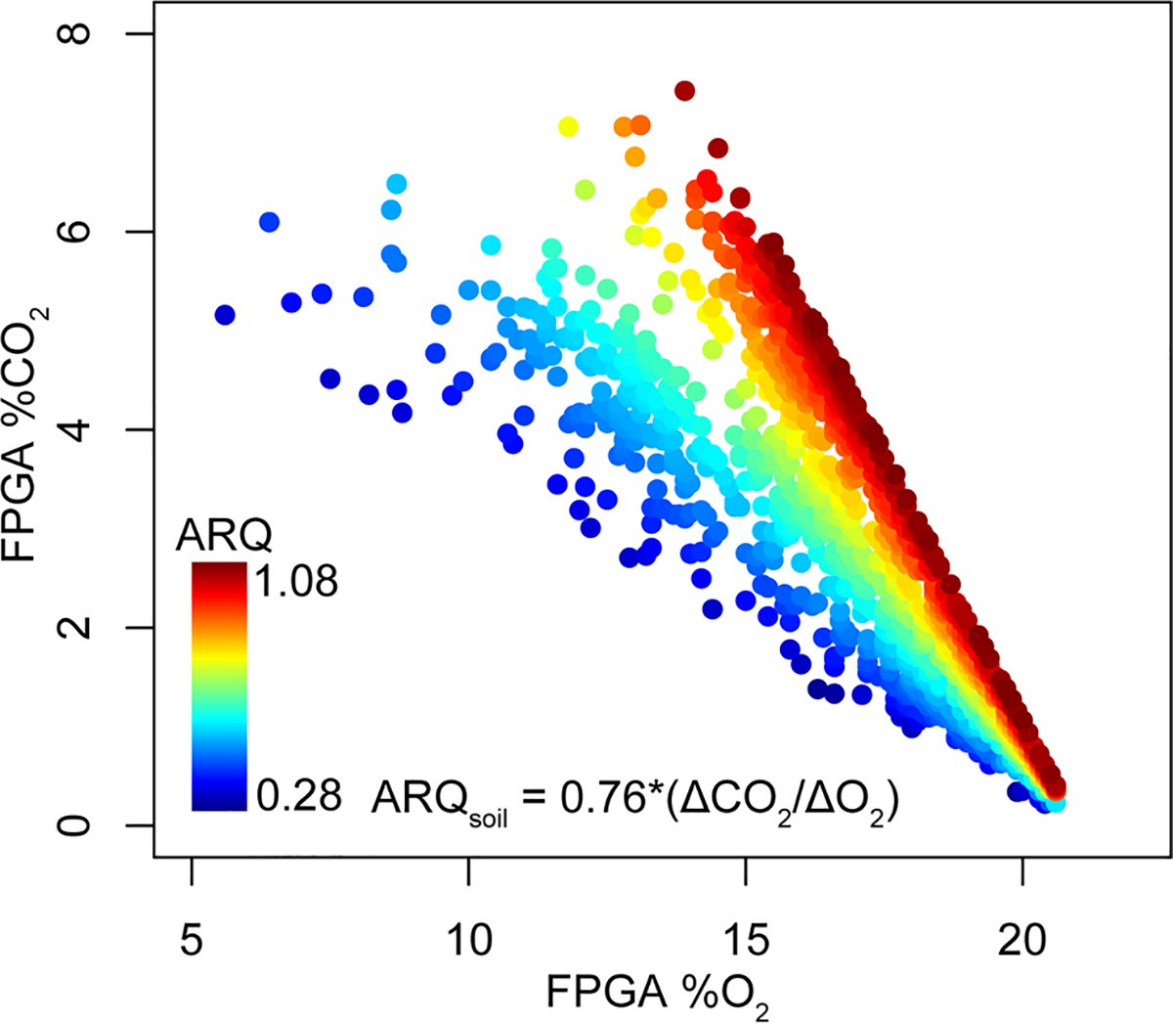
Scatter plot of FPGA-measured CO_2_ and O_2_. Points are colored according to their Apparent Respiratory Quotient values. ARQ values indicate that the apparent consumption of O_2_ is not always balanced 1:1 by observed CO_2_ concentrations in soil profiles. This indicates significant a/biotic interactions for either or both gasses are present in the systems being monitored.

This deviation from 1:1 can be quantified through the calculation of the ARQ which is used to color points in a scatterplot of observed CO_2_ and O_2_ data in Fig 5. This suggests that the FPGA has very high potential to enhance field observations and monitoring of the consumption, production, and transport of metabolic gasses in soils. Because ARQ is a function of the deviation of measured soil atmosphere from ambient aboveground atmospheric concentrations, ARQ calculations from gasses which are very close to ambient concentration in either CO_2_ or O_2_ can be easily skewed by small inaccuracies. A measurement error of ±0.1% CO_2_ is proportionately much greater if the true concentration is 1.1% as compared to 5.1%. Because of this and for illustrative purposes, the plot in Fig 5 omits ARQ values below the 2.5^nd^ quartile and above the 97.5^th^ quartile.

## Discussion

To date, analyses of FPGA measurement data appear in three original research articles including this one [15, 27], with additional manuscripts in preparation. Six FPGAs have been constructed and deployed thus far in the US and abroad, requiring less than $2,000 each in materials, though prices fluctuate. Having made over 2000 soil profile O_2_ and CO_2_ measurements using soil gas wells installed at depth in the soil, FPGAs have proven to be practical for field use requiring hiking on uneven ground over long distances for many hours. Further, comparison to bench-top gas chromatography indicates that FPGA’s accurately measure soil CO_2_ concentrations in the range of 0.1% - 8% observed in the field thus far. Paired with *in-situ* soil O_2_ measurements, nuanced analyses of the data are possible with publicly available temporal weather and satellite imagery data sets. This allows for thorough consideration of apparent soil profile respiration dynamics and allows researchers to incorporate the effects of both biotic and abiotic factors, i.e. a critical zone approach.

While current FPGAs are robust and function reliably in their operation, they are primed for innovation. Areas to be improved include the current use of separate screens for monitoring and data collection of O_2_ and CO_2_ data, the use of a voltmeter for the monitoring of CO_2_ analog data signals, and manual written recording of data measurements. We are currently developing means to directly interface CO_2_ and O_2_ probes with microcomputers and a GUI interface for on-demand recalibration and operation of the FPGA platform as well as automatic calculation of soil ARQ and data export to .csv file. A final additional area of development is the configuration of the FPGA for use in measuring CO_2_ fluxes from the soil surface. We are committed to enhancing the expansibility and modularity of the FPGA platform and receiving feedback from the scientific community on how we can further develop and enhance the utility of these devices into the future.
